# Human Health Risk Assessments of Trace Metals on the Clam *Corbicula javanica* in a Tropical River in Peninsular Malaysia

**DOI:** 10.3390/ijerph18010195

**Published:** 2020-12-29

**Authors:** Chee Kong Yap, Koe Wei Wong, Salman Abdo Al-Shami, Rosimah Nulit, Wan Hee Cheng, Ahmad Zaharin Aris, Moslem Sharifinia, Alireza Riyahi Bakhtiari, Hideo Okamura, Muhammad Saleem, Weiyun Chew, Mohamad Saupi Ismail, Khalid Awadh Al-Mutairi

**Affiliations:** 1 Department of Biology, Faculty of Science, Universiti Putra Malaysia, Selangor 43400, Malaysia; wongkoewei@gmail.com (K.W.W.); rosimahn@upm.edu.my (R.N.); 2 Indian River Research and Education Center, IFAS, University of Florida, Fort Pierce, FL 34945, USA; alshami200@gmail.com; 3 Faculty of Health and Life Sciences, Inti International University, Sembilan 71800, Malaysia; wanhee.cheng@newinti.edu.my; 4 Department of Environmental Sciences, Faculty of Environmental Studies, Universiti Putra Malaysia, Selangor 43400, Malaysia; zaharin@upm.edu.my; 5 Shrimp Research Center, Iranian Fisheries Science Research Institute, Agricultural Research, Education and Extension Organization (AREEO), Bushehr 7516989177, Iran; moslem.sharifinia@yahoo.com; 6 Department of Environmental Sciences, Faculty of Natural Resources and Marine Sciences, Tarbiat Modares University, Mazandaran 46417-76489, Iran; ariyahi@gmail.com; 7 Faculty of Maritime Sciences, Graduate School of Maritime Sciences, Kobe University, Kobe 658-0022, Japan; okamurah@maritime.kobe-u.ac.jp; 8 Department of Chemistry, Government Post Graduate College, Mirpur University of Science and Technology, Mirpur 10250, Pakistan; msaleemqau@yahoo.com; 9 Centre for Pre-University Study, MAHSA University, Selangor 42610, Malaysia; chewweiyun@gmail.com; 10 Fisheries Research Institute, Pinang 11960, Malaysia; saupi@rocketmail.com; 11 Department of Biology, Faculty of Science, University of Tabuk, Tabuk 7149, Saudi Arabia; kmutairi@ut.edu.sa

**Keywords:** human health risk assessment, trace metals, *Corbicula javanica*, Malaysia

## Abstract

This study aimed to analyse ten trace metal concentrations in the edible part of the freshwater clam *Corbicula javanica* and to provide a critical assessment of the potential risks to human health through consumption of this clam as food based on well-established indices and food safety guidelines. The clams were captured from a pristine original site and transplanted to other sites with different environmental qualities. The trace metal levels in the edible total soft tissue (TST) of the clam were below those of the food safety guidelines referred to except for Pb, which exceeded the permissible limit set by the European Commission (2006) and the US Food and Drug Administration/ Center for Food Safety and Applied Nutrition); Interstate Shellfish Sanitation Conference. (USFDA/CFSAN; ISSC) (2007). The estimated daily intake (EDI) values of the clam were found to be lower than the oral reference dose and the calculated target hazard quotient (THQ) and total THQ were found to be less than 1. Therefore, in conclusion, the human health risk for consumption of TST of *C. javanica* at both average and high-level were insignificant regardless of the environment it was exposed to.

## 1. Introduction

Discovery and utilization of trace metal elements have defined the advancement of human civilizations and technologies. However, along with the benefits brought to humans, there are negative consequences of the over-utilization of metals caused by the development of industrial and anthropogenic activities. The worsening of heavy metal contamination in the environment has not only severely affected the production and quality of agricultural crops but has also jeopardized the quality of the atmosphere and the hydrosphere. By the flow of trace metals along the natural food chain, the health of humans as a part of the biota can be negatively impacted in various ways including shellfish consumption [1,2,3,4,5]

Unlike organic pollutants, metal and metal-containing pollutants are chemically non-biodegradable and can be potentially accumulated up to harmful concentrations [6,7]. It has been recognized that environmental contamination by trace metal-containing wastes poses covert, long-term and irreversible threats on the health of humans living in a given environment [6]. Therefore, it is important to determine the health risk assessment of toxic trace metals in this commonly consumed bivalve.

*Corbicula javanica* is a species of freshwater clam that is indigenous to various Southeast Asian countries. There are reports of its presence in Malaysia [8,9,10], Java [11,12,13] and Sumatra [14], in Indonesia and Thailand [15]. It is commonly consumed as food in Situ Gede, Bogor, located in the west of Java Island, Indonesia [12]. It is also considered as a delicacy in the Kelantan state of Malaysia where it is widely consumed as a traditional snack by the Kelantanese who refer to this clam as “Etok” or “Etak” [12,16]. It should be noted that *Corbicula* are hard to distinguished based on their appearance alone. There have been some taxonomic studies suggesting that all *Corbicula* should be synonymized with *Corbicula fluminea*, a well-studied invasive clam species [17,18]. While there is a lack of taxonomic study focused on Corbicula sp. in Malaysia, relevant studies in Thailand have found that the variation of Corbicula there lacks taxonomical significance, and are anatomically, conchologically and genetically indistinguishable [19]. However, due to the existing consensus of scientific reporting in the region, the samples collected in this study will be reported as *C. javanica*.

Due to less industrial development in the Kelantan state, the risk of trace metal contamination is currently not significant. However, it is still necessary to determine the potential contamination of this delicacy in the river. Therefore, in this study, *C. javanica* indigenous to the Langat River was chosen as the study subject and was artificially transplanted from its original pristine habitat to more anthropogenic impacted sites along the river. This was done in order to observe the potential of this clam to accumulate trace metal contaminants in a river stream under a more realistic context instead of under controlled exposure.

Due to its wide distribution and the common consumption of *C. javanica*, it is necessary to access its metal contamination level and its potential risk to human consumers. Human Health Risk (HHR) at the screening level is usually achieved by evaluation of the metal concentration of a food compared to officially sanctioned dietary guidelines. Calculation of the Estimated Daily Intakes (EDI) as well as following the Target Hazard Quotient (THQ) could also provide an objective platform to quantify the non-carcinogenic health risk caused by prolonged consumption of trace metal(s) [20,21,22]. As an integrated risk index, by comparing the concentration of a pollutant ingested with a standard reference dose [21], the index of EDI and THQ had been commonly adopted to assess the HHRs caused by exposure to metal pollutants in polluted food [20,23] and acknowledged as a feasible parameter for trace metal polluted shellfish associated HHR assessment [20,22,23].

The objectives of this study were to determine the concentrations of ten trace metals in the edible part of *C. javanica* and later to make a critical assessment of the potential risks to human health through consumption of this clam as food by using well-established indexes and food safety guidelines.

## 2. Materials and Methods

### 2.1. Study Sites

**Figure 1:**
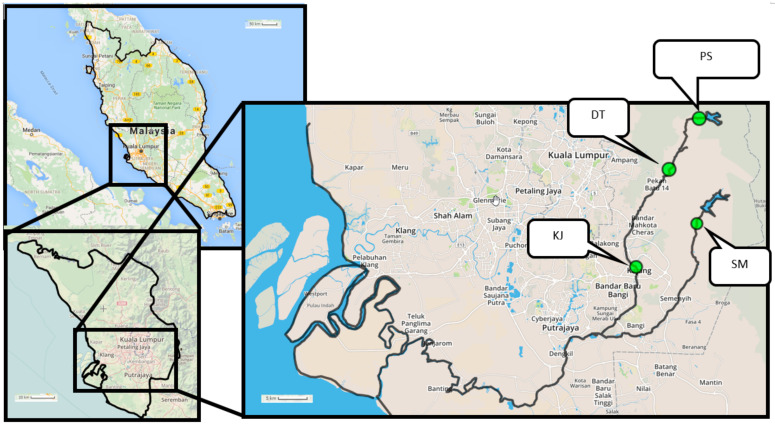
Sampling map for original sites and transplantation sites of *C. javanica* and surface sediment along the Langat River, Selangor, Peninsular Malaysia. (Source: Open Street Map; Drawn with Scribblemap online map drawing tool).

Samplings were carried out between June 2012 and June 2013 at four selected sites located upstream and mid-stream of the Langat River (LR) and upstream of the Semenyih River (SR), a branch of the LR (Figure 1). The description of each of the sampling sites and the sampling map are presented in Table 1 and Figure 1, respectively. The distribution of the clam was concentrated upstream near Kampung Kuala Pangsun, Selangor, where the sandy riverbank can be found. To cope with this problem, the live samples were collected and transplanted to other sites from the sandy upstream of the river. The other sites chosen for this study were based on the perceived levels of artificial inputs of pollutants into the environment.

LR is a 120 km long river on the west coast of Peninsular Malaysia that spans from the Titiwangsa Range in Gunung Nuang and flows westwards toward the Strait of Malacca. Sungai Semenyih and Sungai Labu are two of the major tributaries of the LR. The river basin has an area of about 2423 m^2^ [24]. It is the most important source of freshwater for the population residing in the LR Basin who are concentrated in townships like Bangi, Cheras, Kajang and the Malaysian administrative capital of Putrajaya [25,26]. It is among the most populated and rapidly growing economic regions in this nation. Emissions from the domestic sewage treatment plant in the proximity of LR was identified as a major pollution source for this river, responsible for up to 28% of the total pollutant input. The pollution was strongly associated with anthropogenic activities such as construction, industries and agriculture [26,27]. Major steel production industries as well as other industrial zones located in the proximity of the river are considered a potential source of heavy metal input in this region. [27]. Due to the high population of this region, increasing domestic sewage could soon overwhelm the existing wastewater facilities. The association of reduced biodiversity (low biological monitoring working party (BMWP) score) with reduced water quality, according to Azrina et al. [24] who studied LR, indicated a possible negative impact of anthropogenic activity on the ecosystem. According to statistics from the Department of Environment, Malaysia, LR is listed as “slightly polluted” river (Class III) [28,29].

### 2.2. Three-Day Transplantation, Sample Collection and Field Measurements

In order to minimize the possible variation of *C. javanica* among the local populations, as well as enabling this biomonitoring study to be conducted in locations that were devoid of the clam, individuals from upstream sites were transplanted to downstream sites. The transplanted clam individuals were put into cages for transplantation. These cages were prepared by using a folded 13 cm × 13 cm plastic net tied with cable net. A total of 30 clams for each cage were taken from the upstream site at Pangsun and inserted into the prepared cage. These cages were transported from the Pangsun site to the Batu 16 Dusun Tua, Semenyih and Kajang sites for transplantation. Before the cages were transplanted to the respective sites, the clams in the cages were first acclimatized by soaking the cages in a 50% mixture of water from the original site and the destination site for 30 min. Then the cages were rope tied to a stationary object and gently inserted into the river stream. After the three days exposure period, the transplanted cages were harvested. For the background study, 30 clams were collected at the original Pangsun (PS) site for each sampling session. The collected clams were inserted into an acid-cleaned polyethylene bag and chilled in an ice box to prevent sample degradation during transportation back to the laboratory. The samples were stored at −20 °C until analysis. During the transplantation and the collection of the transplantation cages, several in situ physico-chemical parameters of the river water were also measured and recorded by using a YSI 556MPS handheld multi-parameter instrument (YSI Environmental, OH, USA).

### 2.3. Pre-Treatment of Corbicula Javanica

During the dissection, the thawed clams were removed from the plastic bags and washed with distilled water to get rid of the dirt on the surfaces of the samples. The shell dimensions were first measured and recorded using a Vernier calliper with an accuracy of up to 0.01 cm. The accuracy of the measurement was ensured by re-zeroing after each measurement. After the measurement, the total soft tissue and shell were carefully separated and washed using distilled water. The dissected parts of the clams were individually balanced for its wet weight on a calibrated and re-zeroed electronic balance with an accuracy at 0.0001 g. Weighted parts were pooled and homogenized to obtain enough samples for analysis. The dissected tissues were then dried in an oven at a temperature of 60 °C for 72 h to a constant weight. The final weights were recorded as dry weight. The dried tissue and shell were stored separately inside a dry acid washed plastic bag (Neptune Technology, Malaysia) until analysis.

### 2.4. Microwave Assisted Digestion for FAAS and ICP-MS Analysis

The digestion of the clam samples was performed in accordance with the Milestone Microwave Laboratory System method for oyster tissues. For instance, 0.5 g of dried total soft tissue or shell tissue was digested with 7 mL of HNO_3_ and 1 mL of H_2_O_2_ in a sealed acid-cleaned Teflon vessel in a microwave assisted digester at 200 °C for 25 min. The resulting digestates were diluted to 100 mL with ultrapure water (18.2 MΩcm) by transferring them to a 100 mL acid-washed volumetric flask. Before top-up to the 100 mL mark, the Teflon vessels were rinsed thrice with ultrapure water and the washing water were combined in the volumetric flask. After dilution, the digestates were filtered out by filter paper (Whatman no. 1). The resulting digestates were stored at room temperature in acid-cleaned plastic vessels until used for metal analyses. Heavy metal analyses were done by using Flame Atomic Absorption Spectrometry (FAAS, Perkin Elmer Model AAnalyst 800; Perkin Elmer LLC, CT, USA) for Zn, Cu, Ni, Pb and Fe; and Induced Coupled Plasma-Mass Spectrometry (ICP-MS, Perkin-Elmer Model Elan 600; Perkin Elmer LLC, CT, USA) for Mn, Co, Cr, As and Cd.

### 2.5. Conversion Factor

The conversion factor (CF) was calculated to enable the conversion of dry weight-based data into wet weight-based data for convenient assessment of the impact of trace metal concentration of fresh *C. javanica* on human health. The conversion factor is defined as the ratio between dry weight and wet weight of the soft tissue [10]. The formula for the calculation of conversion factor is as follows

$$Conversion\;factor\;\left(CF\right)=\frac{\mathrm{dry}\;\mathrm{weight}\;\mathrm{of}\;C.javanica\;\mathrm{soft}\;\mathrm{tissue}\;\left(g\right)}{\mathrm{wet}\;\mathrm{weight}\;\mathrm{of}\;C.\;javanica\;\mathrm{soft}\;\mathrm{tissue}\;\left(g\right)}$$

This CF will then be used to mathematically convert AAS measured dry weight-based metal concentration ($Mc_{dry}$) to wet weight-based metal concentration ($Mc_{wet}$) for convenient human health risk assessment that warranted the use of wet weight-based metal concentration.

$$Mc_{wet}=CF\times Mc_{dry}$$

### 2.6. Quality Control and Quality Assurance

All glassware and non-metal apparatuses used in this study were soaked in an acid bath (5% HNO_3_) for 72 h after being washed with laboratory grade detergent (Decon 90; Fisher Scientific (M), Shah Alam, Malaysia), to avoid possible contamination. The metal made apparatuses were washed and soaked in laboratory grade detergent (Decon 90) for at least 3 h before they were used. Procedural blanks were employed, and quality control samples were made by dilutions of the standard solutions of the metals to be tested. These standard solutions were analysed after every 5–10 samples in order to check for the accuracy of the analysed samples [9].

The Certified Reference Material (CRM) was checked with the samples from dogfish liver (DOLT-3, National Research Council Canada) and soil (Soil-5, NSC) to ensure the accuracy of the FAAS and ICP-MS measurements. Their recoveries were acceptable at 73.01–97.07% (DOLT-3) and 86.67–178.13% (Soil-5). The recovery of the CRMs is presented in Table 2.

### 2.7. Statistical Analyses

One-way ANOVA (one-way analysis of variance) and Student-Newman-Keuls (SNK) post hoc analysis were performed using the SPSS software version 21 (IBM, NY, USA) for Windows to determine the differences between the values [30].

### 2.8. Human Health Risk Assessment

Human health risk (HHR) arises due to consumption of clams exposed to toxic metals was evaluated by the mean of the calculation of the Estimated Daily Intakes (*EDI*) and the Target Hazard Quotient (THQ) [31]. The calculation formula for EDI is as followed

(1) $$EDI=\frac{Mc_{wet}\times consumption\;rate}{body\;weight}$$

where $Mc_{wet}$ is the metal concentration (µg/g) in clam soft tissue obtained on a wet weight basis. The body weight for adults was 60 kg and the consumption rate was 17.86 and 35.7 g/day_,_ for average (ALM) and high-level mollusc (HLM) consumers, respectively [32,33]. ALM and HLM are two of the mollusc consumption scenarios proposed by Jović and Stanković [32] in relation to the respective provisional tolerable weekly intake (PTWI) founded by Food and Agriculture Organization/World Health Organization (FAO/WHO) Expert Committee Food Additives (JECFA). They represent two consumption scenarios where consumers consume mollusc by two rates. The oral reference dose (RfD) was compared with the EDIs (µg/kg wet weight/day) of metals in clams. The oral reference dose (RfD) (µg/kg wet weight/day) used in this study were As: 0.30, Cd: 1.00, Co: 30.00, Cr: 3.00, Cu: 40.0, Mn: 140.00, Ni, 20.00, and Zn: 300, as provided by the EPA’s Integrated Risk Information System online database (IRIS) [34]. Since the RfD for Pb was not available according to the EPA’s IRIS (IRIS, 2014), the present study employed the RfD as 3.50 µg/kg wet weight/day as suggested by a former study by Hang et al. [27] and US EPA [34,35]. Since the RfD for Co was not available in the database of the EPA’s IRIS [34], the present study employed the RfD as 30 µg/kg wet weight/day as suggested by a former study by Finley et al. [29]. The oral reference dose is the critical concentration of uptake of a metal, below which there will not be any appreciable risk [31].

In this study, the non-carcinogenic risk assessment on the exposure of trace metal through ingestion of the clams was determined through the calculation of *THQ*. *THQ* is defined as a ratio between the estimated dose of a trace metal and the oral reference dose. The *THQ* was calculated with the formula defined by US EPA [35].

(2) $$THQ=\frac{EF\times ED\times CR\times Mc_{wet}}{RfD\times BW\times AET}\times 10^{-3}$$

where *EF* is exposure frequency (365 d/year); *ED* is the exposure duration (70 years), equivalent to the average lifetime; *CR* is the consumption rate (17.86 and 35.7 g/d for average and high level mollusc consumers, respectively [25]; *Mc* is the metal concentration in clam (µg/g wet weight); *RfD* is the oral reference dose as cited above; *BW* is the average body weight (60 kg for adults); *AET* is the average exposure time for non-carcinogens (365 d/year × *ED*); and 10^−3^ is the unit conversion factor.

According to US EPA [17], the value of THQ above 1 (THQ > 1) indicates that the exposed population is likely to experience obvious deleterious non-carcinogenic effect via the consumption of metal contaminated foods. The total THQ is calculated based on the summation of the THQ values of all metals.

## 3. Results and Discussion

### 3.1. The Allometric Parameters of Clams

The mean values of the lengths, widths and heights of the shells of *C. javanica* as well as the fresh weights and dry weights of the shells and the total soft tissues of *C. javanica* are shown in Table 3. For all the 735 individuals captured from site PS, the values (mean ± SD) of the length, width and height of *C. javanica* and the total fresh weight were 15.65 ± 2.42 mm, 12.86 ± 1.93 mm, 8.26 ± 1.31 mm and 0.54 ± 0.27 g, respectively.

The three dimensions of *C. javanica* measured in this study were similar to those reported by Yap and Mohd Khairul [36]. The mean values for the allometric parameters of *C. javanica* by Yap and Mohd Khairul [36] were 14.95–20.29 mm for shell length, 8.14–11.23 mm for shell width and 12.20–16.87 mm for shell height. For the water content of the total soft tissue, the mean water content found in this study was 88.63%, similar to the range of the mean value of total soft tissue water content of 85.54–89.03% found by Yap and Mohd Khairul [36].

*Corbicula japonica*, a genetic siblings of *C. javanica*, were studied by Izumi et al. [37] as a bioindicator for protozoan *Cryptosporium parvum* oocyst in blackish water of the Ishikari River, Japan. The body sizes of *C. japonica* were 33.33–43.1, 29.6–37.1, 18.0–24.1 mm with body weights of 15.5–16.5 g. As indicated from the study of Izumi et al. [29], *C. japonica*’s size was generally larger than that of *C. javanica*. The body weight of *C. japonica* was also much heavier than that of *C. javanica*.

*Corbicula fluminea*, another genetic sibling of *C. javanica*, is regarded as an invasive species in the rivers and lakes of five continents [38,39,40] and is considered as one of the most efficient freshwater invaders worldwide [38]. Xiao et al. [40] studied the effects of temperature and salinity on the metabolic rate of *C. javanica*. The mean size (small size/medium size/large size, mm) of their findings on the sizes of *C. fluminea* were 19.31/24.24/28.19, 17.70/22.07/25.51 and 12.88/15.09/17.08 for shell length, shell height and shell width, respectively. This finding of Xiao et al. [40] on the size of *C. fluminea* showed that *C. javanica* was of similar size. The mean dry weights (small size/medium size/large size, g) of *C. fluminea* measured by Xiao et al. [40] were 0.526/0.903/1.310 and 7.684/15.132/21.847 for the soft tissues and shells, respectively.

Compared with the dry weights of *C. javanica* measured in this study (mean dry weight 0.03 g for total soft tissue and 0.44 g for shell), despite being similar in size, the dry weights of both the soft tissues and the shells of *C. fluminea* were higher than those of *C. javanica*. In this study, the tissue water content of *C. javanica* after transplantation from PS to KJ decreased from 89.99% at PS to 86.47% at KJ (Table 3) This might be the result of the elevated salinity in that site (0.01 ppt at PS to 0.08 ppt at KJ, Table 4). Hosoi et al. [38] also revealed that the tissue water content of blackish water living *Corbicula sandai* decreased from 86.3% in freshwater to 88.0 and 85.2% after exposure to 0.1% and 0.3% hypersaline water.

At the transplantation site KJ, the clams showed slightly lower water content (Table 3), reduced slightly from 9.06 ± 4.83% at the original site PS to 7.66 ± 2.56% in KJ. For site DT, the water content in the shell increased to 10.80 ±2.55% while it was reduced to 7.83 ± 2.69% at site SM. The total soft tissue also showed lower water content after transplantation. The water contents of the total soft tissues were reduced from 89.99 ± 2.77% to 88.81 ± 2.23%, 88.72 ± 2.71% and 86.47 ± 3.35% for sites DT, SM and KJ, respectively (Table 3). The association of the metal stress with the water content of an organism was studied by [41] who found that the water content of wheat plant tended to decrease along with the increase of the exogeneous metal stress. A previous study using *Corbicula* spp. exposed to sublethal dose of contaminant showed that *Corbicula* spp.’s tissue water content was increased following the exposure of the clam to asbestos [42] and sodium dodecyl sulphate [43]. The results of the current study and their comparisons to those of previous studies showed that the water content in an organism responded according to the environmental stress experienced.

Aquatic invertebrates lack advanced homeostatic mechanisms to maintain their internal osmolality [44]. Thus, it is implied that the water content and the dry to wet ratio of the soft tissue of an aquatic invertebrate like *C. javanica* could reflect the geo-chemical composition of its natural habitat.

According to Mo and Neilson [45] the use of dry weight rather than wet weight as a measurement of body size of oysters has been recommended by numerous authors due to the high seasonal differences of wet weights and the large differences in estimates among the methods employed in wet weight measurement. Kremer et al. [46] also found that the standardization of wet measurements was difficult even in laboratory settings. Therefore, all metals were analysed on the dry weight basis in this study. However, there are still some metal data presented on a wet weight basis and the safety guidelines of foods and various health assessments are still based on wet weights. A conversion factor (CF), which is the ratio of dry weight versus wet weight, is used to convert dry weight-based data into wet weight for the calculation of human health assessment indexes such as EDI and THQ.

### 3.2. Trace Metals in Transplanted Clams

The trace metal concentrations (µg/g dry weight) in TST and Shell (Sh) are presented in Table 5. After three days of exposure, the As concentration increased from 2.33 at PS to 10.5 at SM, followed by KJ and DT. Cd concentration in TST after 3 days of transplantation increased slightly from 0.19 at site PS to 0.26 at site SM and 0.21 at KJ, and site DT (0.20). Increases in concentration occurred for Co after 3 days of exposure at the transplantation site. The Co concentration increased from 0.56 at the original site to 0.74 at site SM, 0.68 at site DT and 0.63 at site KJ. For Cr, its concentration in the TST of *C. javanica* collected at site PS (4.29) decreased after 3 days of exposure at site SM (3.31), DT (2.72) and KJ (3.44). For Cu, the concentration increased from 16.2 at site PS to 18.2 at site DT and 17.1 at site KJ, while the Cu concentration in site SM decreased to 15.99. For Mn, a decrease occurred after 3 days of transplantation. The Mn concentration decreased from 4.08 at the original site PS to 3.79 at site SM, 2.45 at site DT and 2.96 at site KJ. For Ni, the concentration increased slightly from 13.3 at original site PS to 13.7 at SM where the highest level of Ni was found, while a decrease of Ni concentration was found at sites DT (10.3) and KJ (11.6). The Fe concentration showed a descending trend from the upstream site PS (2335) to lower stream sites SM (1523), DT (1748) and KJ (1018). After 3-day transplantation, the Pb level increased from 10.2 at site PS to 15.8 at site SM, 17.0 at site DT and the highest Pb level was found in site KJ at 27.4. After transplantation, the Zn concentration in the clams decreased from 180 at PS to 149 at site SM, 142 at site DT and 114 at site KJ.

The As in the TST of *C. javanica* at SM was significantly higher (*p* < 0.05) than at the other sites (Table 5). Although the As levels in the sediments in PS and KJ were not significantly different (*p* > 0.05) [47], the As in the TST showed a significant difference (*p* < 0.05). This might be due to possible higher bioavailable As in KJ than in PS. This was supported by the higher As in the non-resistant fractions of the sediment from KJ [41]. The non-resistant As in the sediment peaked at SM [41], in agreement with the peak of As in the TST at SM. The sediment Cd in SM was significantly (*p* < 0.05) higher than in the rest of the sites and so was the Cd in the TST [47]. This showed that the sediments in SM contained more bioavailable Cd [41] as evidenced by the peak non-resistant fraction in SM.

The levels of Co, Cr, Fe, Mn, Ni and Zn in sediment were significantly higher (*p* > 0.05) at PS compared to SM, DT and KJ [47]. However only Cr, Fe and Zn in TST from PS were significantly higher than at the other sites, suggesting that the Cr, Fe and Zn levels in the TST were affected by the respective metal levels in the sediment [41]. The Mn levels in the sediment and TST were peaked at PS as well [47]. Despite the significant differences (*p* > 0.05) in Mn levels in the sediments between PS and SM [47], the Mn levels in the TST between PS and SM were not significantly different (*p* < 0.05). For Co, the high levels in the sediments were not reflected its levels in the TST [47]. For Ni, the highest levels in the sediments and TST were found in PS [47].

The highest levels of Pb and Cu in sediments were found in KJ [47]. However, there was no correlation between the Cu levels in the TST and in the sediment [47]. When Joy et al. [48] exposed *Corbicula* spp. to determine the amount of Cu in an artificial stream for 9 weeks, they found that there were only low variations among the biweekly samples. Harrison et al. [49] conducted a flow through study on *Corbicula* spp. where the clams were exposed to 230, 102, 56, 25 and 11 µg/L of CuCl_2_ through water for 14–35.3 days. They found that the tissue concentrations of *Corbicula* spp. were 16.70, 45.50, 35.20 and 10.90 µg/g dry weight, respectively. This showed that the TST of *Corbicula* sp. might not be able to accurately reflect the Cu levels in the surrounding environment. The Pb levels in the TST of *C. javanica* were positively correlated with the Pb levels in the sediment [47]. This result was in agreement with those of Yap et al. [50] who found that the Pb in the TST of *Perna viridis* had positive correlation (*p* < 0.05) with the Pb in the sediment. Marasinghe Wadige et al. [51] studied the effect of Pb-spiked sediments on the freshwater bivalve, *Hyridella australis.* They found that a spike in Pb concentration in the sediments in laboratory conditions was able to cause an increase of Pb concentration in the soft tissues of *H. australis*.

### 3.3. Comparison of Trace Metal Levels with Reported Studies

The trace metal concentrations in the TST of the *C. javanica* are compared with those in other mollusc species from other regional studies in Tables 6 and 7. The intake of heavy metals by a mollusc species may well be influenced by several factors including the specificity of the species and habitat. Due to shear difference between the context of different studies, the comparison between studies with different species and contexts should be taken with caution. However, it could still give us an objective idea of the bioaccumulation potential of a mollusc species. Several previous studies also included this comparison in their reporting [52,53,54,55].

One of the earliest uses of molluscs as a bio-monitor was that of the marine green mussel *P. viridis* by Yap et al. [50] who later also used the mangrove snail *Telescopium telescopium* [56] and the mangrove snail *Nerita lineata* [20]. Levels of metals in other gastropods were also studied in Argentina [57], Portugal [58], Russia [59,60], Japan [59], China [59,61], South Korea [59], France [62], Antarctica [63], Austria [64], Italy [3] and India [65] (Table 6 and Table 7).

The As concentrations in the clams were found to be different from those in species sibling *Corbicula fluminea* [58]. According to that study, the As concentrations (µg/g dry weight) in the soft tissue of *C. fluminea* (10.80) were higher than the 4.51 found in the current study. This might be due to the different capabilities of pollutant accumulation and metabolism rate between these two species. Since there was no previous controlled laboratory study on the *C. javanica* accumulation rate for As in TST, further studies should be done to verify this hypothesis. Compared to the TST of *N. lineata*, an estuary snail found in the coastal area of Peninsular Malaysia, the As concentrations in it were similar to those of the current study.

The Cd concentrations in the clams were found to be similar to those of the soft tissue of its freshwater living invasive sibling, *C. fluminea* by Villar et al. [57], but were lower than those of its gills (fresh weight-based concentration) as reported by Achard [62]. Since Achard [62] recorded the metals concentration on a fresh weight basis, their findings were not necessarily comparable. When compared with *C. japonica*, which is a blackish water sibling of *C. javanica*, the gill of *C. japonica* had higher Cd concentrations than *C. javanica*. This might be either because of the fact that the *C. japonica* was living in an environment vastly different from freshwater living *C. javanica*, or the possibility that Cd might have a tendency to accumulate in the gill part of the soft tissue. Macías-Mayorga et al. [66] studied the relationship between oxidative stress and Cd accumulation in the mollusc *Crassostrea angulate.* They found that the Cd concentrations in the gill were higher than that of the digestive gland, indicating differences in metal accumulation capabilities among organs. Vodopivez et al. [63] also found that the Cd levels in the gill, digestive gland and kidney of the saltwater mussel *Laternula elliptica* were different, further indicating that the metal accumulations in the soft tissues might not be even. Differences in taxonomy might have contributed to the differences in Cd accumulation, too. Further research is needed to clarify. Gundacker [64] compared metal bioaccumulations in the freshwater molluscs of an urban river habitat in Vienna. The species studied were *Anodonta* sp. and *Unio pictorum*, both freshwater mussels. The Cd levels in various parts of *Anodonta* sp. and *Unio pictorum* were in agreement with those of *C. javanica,* with lower concentrations in the shells (not reported) and higher concentrations in the soft tissues. The Cd in all types of soft tissues of these two mussels were evenly distributed and its concentration was similar with that of *C. javanica*. Compared with other species, the Cd concentrations in *C. javanica* were lower than in *Radix ovata* [64], *Onchidium struma* [61] and *P. viridis* [50], but lower than in *Viviparus* sp. [61] (Table 6).

Compared to *Villorita cyprinoides* living in the coastal region of the Cochin backwaters, India (Table 7), the Co concentration of this marine bivalve was found to be much higher than the Co concentration in the TST of *C. javanica*. For Cr, *Laternula elliptica as* studied by Vodopivez et al. [63] showed similar total Cr concentrations in all organs. *Nerita lineata* studied by Cheng and Yap [20] also showed similar Cr concentrations compared to *C. javanica* in this study. *Onchidium struma* in the study of Li et al. [61] had Cr levels in the soft tissues of the snail that were higher than those of *C. javanica*.

Compared to *C. fluminea*, the Cu concentrations in *C. javanica* were found to be lower than in this invasive sibling, but its Cu levels were within the range of the soft tissue of *Limnoperna fortunei* [57]. In contradiction to the findings of Villar et al. [57], Achard [62] found that the fresh weight-based Cu concentrations in the soft tissue of *C. fluminea* in France were much higher than those of Villar et al. [57] and of the current study. The Cu concentrations in *C. javanica* were also lower than the total Cu in the soft tissue of *Laternula elliptica.* The *C. javanica levels* were similar to those of *Nerita lineata*. As for *Anodonta* sp. and *Unio pictorum*, the Cu concentrations in the soft tissues of these two freshwater mussels were generally higher than those of *C. javanica* found in this study. However, both of these two freshwater mussels contained higher Cu concentrations in the soft tissues and lower levels in the shells (not reported), in agreement with this study. Freshwater snails like *Radix ovata* and *Viviparus* sp. were found to have higher Cu contents in their soft bodies than *C. javanica*. Estuary snails such as *Onchidium struma* and *Telescopium telescopium* are also able to accumulate much more Cu than *C. javanica*. Compared to a previous study of *Perna viridis* by Yap et al. [50], the Cu concentrations in *C. javanica* were within the range of *P. viridis*.

The Fe levels in the clams of this study were found to be higher than those reported by many previous studies such as that on the estuary snail *N. lineata* and *Onchidium struma*, except for *Laternula elliptica,* where the total in the soft tissues was higher than that of *C. javanica*. These differences in Fe accumulation might be due to differences in habitats. However, even species that are closely related, or even the same species, may have differences in metal concentrations in different types of tissues [67].

The mean (µg/g dry weight) of Pb in *C. javanica* was 18.87, higher than the whole range (min–max, µg/g dry weight) of the soft tissues of *Anodonta* sp. (0.10–1.99, 1.09–21.30, 0.16–3.18, 0.12–0.98 for viscera, gill, mantle, abductor muscle, respectively) except for the maximum value of 21.30 µg/g dry weight in the gill of *Anodonta* sp. The same also applied to *Unio pictorium,* where the range of Pb levels in the viscera, gill, mantle and abductor muscle were 0.33–0.57, 1.13–4.68, 0.29–2.20 and 0.38–0.88, respectively. All of these values were lower than those of the current study. Besides, the Pb levels in *C. javanica* were also higher than the mean or range of the soft bodies of *Radix ovata* (1.5), *Viviparus* sp. (1.37), *Onchidium struma* (hepatopancreas, muscle, albumen gland, vitelline gland and digenetic gland, 1.23–1.95, 0.93–1.04, 0.78–0.98, 0.92–1.28, 0.55–0.83, respectively), the soft tissue of *T. telescopium* (4.7) and TST of *P. viridis* (2.0–8.76).

The Zn levels in *C. javanica* of 146.31 µg/g dry weight, fell within the range of Zn in *C. fluminea* (117–163) and were a little higher than those for *Limnoperna fortunei* (48–133). The Zn levels in the gill and digestive gland of *Laternula elliptica* were lower than those in *C. javanica* while its kidney contained Zn at a much higher level than in *C. javanica* in this study. When compared with *Anodonta* sp. and *Unio pictorium*, the Zn levels in *C. javanica* were lower than the summation of all soft tissues (viscera, gill, mantle and abductor muscle). The Zn levels in *C. javanica* were also lower than those in the soft bodies of *Viviparus* sp. The Zn levels in *T. telescopium* were lower than those in *C. javanica* and within the range of those in *P. viridis*.

From the comparisons of Table 6 and Table 7, we can see that the metals in other species from close sibling of *Corbicula* sp. to snails, are greatly different. These differences might have been caused by differences in the contexts of the studies as well as their possible different metabolisms regarding the metal pollutants. Future studies are recommended to uncover the molecular mechanisms of metal metabolism in molluscs by using multiple “omics” approaches.

### 3.4. Comparisons of Trace Metal Concentrations in Established Food Safety Guidelines

Table 8 shows the trace metal concentrations (converted to µg/g wet weight) of TST of *C. javanica* from the present study. The range of wet weight-based concentrations of Zn, Cu, Pb, Ni, Fe, Mn, Co, Cr, As and Cd ranged from 13.87–19.61, 1.49–2.56, 1.12–4.11, 1.04–1.93, 48.89–597.53, 0.23–0.56, 0.05–0.16, 0.29–0.54, 0.15–0.95 and 0.016–0.037, respectively. In general, the concentrations of these metals in *C. javanica* were as follow Fe > Zn > Pb > Cu > Ni > As > Mn > Cr > Co > Cd. The food safety guidelines for trace metals set by different organizations or countries are shown in Table 9.

Zn is an essential trace element that can be toxic to aquatic biota [74] but is present in all organisms for metabolic processes [20,75]. In this study, the Zn levels in the *C. javanica* tissue ranged from 13.87–19.61 µg/g wet weight. The present Zn ranges were within the permissible limits suggested by Food Safety and Standard (contaminants, toxins and residues) Regulations 2011 (India) [72] and Malaysian Food Regulation [73].

Cu is also an essential element for different enzymes in all organisms and is important in the synthesis of haemoglobin [76]. Despite this fact, it can still be toxic to aquatic biota at elevated concentrations [74] and adverse effects are expected at high levels of human consumption [20]. In this study, the ranges of Cu in *C. javanica* were 1.49–2.56 µg/g wet weight. The present Cu ranges were all below the permissible limits suggested by Food Safety and Standard (contaminants, toxins and residues) Regulations 2011 (India) [60] and Malaysian Food Regulation [73].

Pb is a non-essential toxic metal that causes many adverse health effects such as neurotoxicity and nephrotoxicity in elevated concentrations [20,74,77]. In this study, the range of Pb concentrations in soft tissues of *C. javanica* was 1.12–4.11 µg/g wet weight. The Pb concentrations of the soft tissues of *C. javanica* at sites PS, SM and DT were below the permissible limits suggested by the Australia New Zealand Food Standard Code [68], Food Adulteration (metallic contamination) Regulation, Hong Kong SAR [69], Ministry of Health and Family Welfare, India [60] and Malaysian Food Regulation [61]. The Pb concentrations from sites SM and DT were found to exceed the permissible limits suggested by Commission Regulation (EC) No 1881/2006 (European Union) [70] as well as by USFDA/CFSAN; ISSC, United States [71]. The Pb concentration in the soft tissue of *C. javanica* in KJ was found to be 4.11 ± 2.18 µg/g wet weight, which was higher than all permissible limits mentioned above.

The Fe concentrations in the *C. javanica* soft tissue ranged from 48.89–597.53 µg/g wet weight. Although abundant in the environment and considered to be an essential element, acute Fe overload is potentially life threatening. Chronic Fe overload leads to (in the extreme, lethal) damage of organs such as heart and liver [20]. However, the nature of the accumulated damage that results in such organ failure is not yet fully known [78].

The Cr concentrations in *C. javanica* ranged from 0.29–0.54 µg/g wet weight. According to Nordberg et al. [68], Cr is considered as a pollutant, but it is also a micronutrient and its usable form plays an important role in glucose metabolism. In this study, all sampling sites were found to be lower than the allowable limit for fishery products by the Food Adulteration (metallic contamination) Regulation, Hong Kong SAR [69], and USFDA/CFSAN and ISSC, United States [71].

The range of As concentrations in *C. javanica* was 0.15–0.95 µg/g wet weight. As is a semi-metallic element which is a potent toxin and carcinogen and is considered to be an environmental pollutant due to the significant input of industrial activities [20,79]. Its toxicity is largely dependent on its chemical forms and it is only considered toxic if present in inorganic forms, such as arsenate and arsenite [80]. In this study, the As concentrations of the soft tissues of *C. javanica* from all sites were found to be lower than all of the allowable levels shown in Table 9.

Cd is a non-essential element for organisms and is considered as a highly toxic metal to the biota and teratogenic and carcinogenic to humans [20,81]. The Cd concentrations in *C. javanica* were 0.016–0.037 µg/g wet weight. None of the sampling sites were over 1.0 µg/g wet weight. Therefore, none of the sites exceeded the permissible limit of 1.00 µg/g wet weight by World Health Organization [82] and Malaysian Food Regulations [73], the 1.5 µg/g wet weight by [60], the 2.0 µg/g wet weight by Food Standards Australia and New Zealand [68] and by Food Adulteration (metallic contamination) Regulation, Hong Kong SAR [69], and lastly the 4 µg/g wet weight by USFDA/CFSAN; ISSC, United States [71].

### 3.5. Estimated Daily Intake

The EDI of trace metals through *C. javanica* by average (ALM) and high-level mollusc (HLM) consumers are listed in Table 10. The EDI values (µg kg wet weight/day) for ALM consumers for all sampling sites were 0.007–0.038 for As, 0.006–0.015 for Cd, 0.018–0.028 for Co, 0.089–0.154 for Cr, 0.530–0.761 for Cu, 45.5–76.5 for Fe, 0.080 to 0.135 for Mn, 0.337–0.583 for Ni, 0.332 to 1.223 for Pb and 4.638–5.897 for Zn. The EDI values for HLM consumers for all sampling sites were 0.015–0.075 for As, 0.013–0.031 for Cd, 0.037–0.056 for Co, 0.178–0.307 for Cr, 1.059–1.522 for Cu, 90.8–152.8 for Fe, 0.160–0.271 for Mn, 0.674–1.165 for Ni, 0.664–2.445 for Pb and 9.272 to 11.787 for Zn.

The EDI values in both ALM and HLM consumers were lower than RfD values (Table 10). Therefore, this strongly indicated that the consumers would not experience any significant health risks from the intake of all the metals studied through the consumption of *C. javanica.*

EDI has been widely used by multiple studies aiming to assess human health risk. The values of EDI found in this study are compared with others’ findings across the globe in Table 11. The EDIs of As, Cd, Co, Cr, Cu, and Mn in the current study were found to be lower than the EDI values of other species. The EDI of Ni from this study was higher than those of all of the species referred to in Table 11, except for *Venerupis rhomboids* [83] and *Donax trunculus* [3]. The EDI of Pb from the current study was to be found lower than those of *N. lineata* [20], *Ruditapes decussates, Mactra coralline, Paphia undulate* and *Coralliophila meyendorff* [83], but higher than those of *Venerupis rhomboids, Crista pectinate* [83], *Ostrea plicatula, R. philippinarum, Sinonovacula constricta, Tegillarca granosa* [84], and *D. trunculus* [3]. The EDI of Zn in this study was lower than those of *Crassostrea angulate, C. hongkongensis* [85] and *D. trunculus* [3], but comparable with that of *N. lineata* [20].

The overall EDI values of *C. javanica* found in this study were different from those of other species mentioned above which suggested that these species had different bioaccumulation capabilities This might be due to the differences in their natural habitats. There must have been a difference in their respective trace metal sources. The differences in their abilities to metabolize the metal pollutants accumulated in their bodies might also have contributed to the differences in the EDIs. Metallothioneins (MT) are non-enzymatic proteins with low molecular weights, high cysteine contents, non-aromatic amino acids and good heat stabilities [87]. They are regarded as being central constituents of metal metabolisms. Beg et al. [88] measured the metallothionein in the gills and livers of the demersal and pelagic fish species. Their study supported our hypothesis that the rates of metal metabolism (metallothionein) of different species are different.

### 3.6. Target Hazard Quotient

The THQ values of all the metals are presented in Table 10. All of the THQ values are scored below 1 for all metals through consumption of *C. javanica* from LR. This indicated that the health risks associated with trace metal exposure for ALM and HLM were insignificant. The ranges of total THQ values (value × 10^−5^) for ALM consumers and HLM consumers were 24.0–54.0 and 48.1–108, respectively. The total THQ values for all sites and all metals for both ALM and HLM consumers all scored below 1, suggesting that the risk of trace metal intake by consumption of *C. javanica* for both ALM and HLM consumers was low.

The averaged values of THQ of all sampling sites in this study were compared with other international findings in Table 12. The THQ values of As, Cd, Co and Pb of the current study were found to be lower than the THQ values of other species. The THQ of Cr from the current study was lower than of *N. lineata, R. decussates, Mactra coralline, Paphia undulate, Ostrea plicatula, R. philippinarum*, *Sinonovacula constricta* and *Tegillarca granosa*, while the THQ of Cr from the current study was comparable to those of *Coralliophila meyendorff, Venerupis rhomboids* and *Crista pectinate*. The THQ of Cu of *C. javanica* was comparable to that of *N. lineata*, but lower than those of *C. angulate and C. hongkongensis* (Table 12). The THQ of Ni from this study was higher than all of the species referred to in Table 12. The THQ of Zn was higher than that of *N. lineata,* but lower than those of *C. angulate* and *C. hongkongensis*.

Otachi et al. [77] reported that elevated Cd, Li, Sr, and Zn levels with high THQ (THQ > 0.1) suggested potential risk for consumption of the fish muscle of *Oreochromis leucostictus* collected from Lake Naivasha, Kenya. Otachi et al. [89] also reported high THQ for the tissues of the fish *Hydrocynus forskahlii* (Cuvier 1819) from Lake Turkana, Kenya suggesting health risk for consumption of the said fish. Iwegbue [90] determined the THQ for Cd, Pb, Ni, Cr, Cu, Co, Fe, Mn and Zn of selected brands of canned mackerel, sardine and tuna in Nigeria. With the estimated THQ found to be lower than 1 for the majority of samples, it was concluded that there was no long-term health risk for the consumption of the said food. Han et al. [91] described the impact of metal pollution in seafood and assessed the potential health risk from consuming contaminated oyster (*C. gigas*) in Taiwan. They found that 50% (12 of 24 THQs) of the THQs exceeded 1 for the maximally exposed individuals consuming oysters collected from Machu Island, Taiwan. This suggested that long-term exposure to four metals (Cu, Zn, Cd and inorganic As) through consumption of oysters would have potential health risks, especially for the Machu Islands area. Cheng and Yap [20] determined the THQ value for As, Cd, Cr, Cu, Hg, Pb and Zn for the mangrove snail *N. lineata* collected from various sites from Peninsular Malaysia. Cheng and Yap [20] found that the calculated THQs were less than 1 but the total THQ from all the sites were found to be more than 1 for high level consumers except for one single site. Therefore, Cheng and Yap [20] suggested that moderate consumption of *N. lineata* is advisable to avoid health risks to consumers. Li et al. [84] reported that the total THQ based on Hg, Pb, Cr and Cd, for all four shellfish were below 1, indicating that the intakes of trace metals by consuming these shellfish collected from Xiamen, China, did not result in an appreciable hazard risk to the human body.

### 3.7. Potential Mitigation Measures to Combat Public Trace Metal Hazard

Overdoses of heavy metals are toxic, mutagenic, teratogenic and carcinogenic to the human body [92]. Additionally, these elemental pollutants are known to have biomagnification potentials [93,94]. Their potential threat should not be ignored and a coordinated strategy must be formed to counteract potential trace metal toxicity in food not only in Peninsular Malaysia but also in other directly or indirectly related geographical regions.

Application of antioxidant nutraceutic may be a potentially effective way to mitigate chronic trace metal hazard through oral ingestion. Based on previous in vitro and in vivo studies, several natural products have been suggested to have the potential to mitigate heavy metal toxicity. Vitamin E has been demonstrated to be able to reduce Cd accumulation in the kidney, liver and blood of rats, as well as to fortify antioxidant levels, reduce lipid peroxidation and increase cell viability [95]. Vitamin E has also been proven to have the ability to reduce the oxidative damage on delta-amino-levulinic dehydratase induced by lead intoxication in rat erythrocytes [96]. The antioxidant effect of vitamin E also been observed to protect against heavy metals (Pb, Hg, Cd and Cu) indicated in renal and testicular oxidative stress and injuries in male mice [97].

In addition to Vitamin E, Curcumin [98,99,100,101,102], N-acetylcysteine [103,104,105,106], α-lipoic acid [107,108,109], melatonin [110,111,112,113,114,115], flavonoid [116,117,118], anthocyanidins [119], quercetin [120], naringenin [121], black tea [122], olive oil [123], and sesame oil [124] have been discovered to have beneficial effects on battling trace metal induced toxicity in various in vitro and in vivo studies. However, it should be noticed that most of the studies referred to here are in fact in vitro or in vivo studies that utilized a cellular and animal biological model. None of these studies directly indicated that the same protective effect will be replicated in a human context. Human trials will be needed to confirm the protective effect on human health. Besides the application of natural products mentioned above, the introduction of an optimum amount of nontoxic chelating agents may also help in mitigating trace metal risks [114,125,126].

## 4. Conclusions

This human health risk assessment was based on EDI, THQ and total THQ via the consumption of the clam *C. javanica* collected from the transplantation study in the LR. The trace metal levels in the TST of *C. javanica* were all below the food safety guidelines, with Pb as an exception. The Pb concentrations from sites SM, DT and KJ were found to exceed the permissible limits suggested by the European Commission [70] as well as by USFDA/CFSAN; ISSC, USA [71]. However, the EDI of *C. javanica* were found to be all lower than the oral reference dose (RfD) guidelines for all metals. Furthermore, the calculated THQ and total THQ were found to be less than 1. Therefore, there was no human health risk based on both the average and the high-level consumption of the total soft tissue of *C. javanica* in terms of THQ.

## Figures and Tables

**Table 1:** Site description for sampling of *Corbicula javanica* collected from the upstream site and transplanted to downstream sites.

Sites	Sites	GPS	Site Description
PS	Pangsun (upstream)	3°12′37.2″ N 101°52′39.6″ E	Upstream of Langat River. Remote village with few residences, near the Pangsun Dam.
SM	Semenyih (upstream)	3°3.382′ N 101°52.411′ E	Sungai Tekala which is a branch of Semenyih River which in turn is a branch of Langat river. Remote village with few residences, near Semenyih Dam and downstream of Tekala River Recreational Area. Palm oil plantation within walking distance.
DT	Kampung Dusun Tua (downstream)	3°08′09.1″ N 101°49′59.9″ E	Small size township
KJ	Kajang (downstream)	2°59.657′ N101°47.063′ E	Mid-size township at midstream of the river, significant human activities.

**Table 2:** A comparison of the measured and certified values (μg/g dry weight) for the heavy metals Zn, Cu, Ni, Pb and Fe for soil (Soil-5, International Atomic Energy Agency, Vienna, Austria) and DOLT-3 (dogfish liver, National Research Council Canada).

Metal	Certified Reference Material (CRM)	Certified Value	Measured Value	Recovery (%)
Zn	Soil-5 (Soil)	680	1211.29	178.13
	DOLT-3 (Dogfish liver)	86.6	80.94	93.46
Cu	Soil-5 (Soil)	21	18.2	86.67
	DOLT-3 (Dogfish liver)	31.2	30.28	97.07
Ni	Soil-5 (Soil)	20.4	23.65	115.95
	DOLT-3 (Dogfish liver)	NA	NA	NA
Pb	Soil-5 (Soil)	98	100.68	102.73
	DOLT-3 (Dogfish liver)	NA	NA	NA
Fe	Soil-5 (Soil)	NA	NA	NA
	DOLT-3 (Dogfish liver)	1484	1083.47	73.01
Mn	Soil-5 (Soil)	1760	1552.5	88.21
	DOLT-3 (Dogfish liver)	NA	NA	NA
Co	Soil-5 (Soil)	14.2	12.78	90
	DOLT-3 (Dogfish liver)	NA	NA	NA
Cr	Soil-5 (Soil)	62	54.81	88.4
	DOLT-3 (Dogfish liver)	NA	NA	NA
As	Soil-5 (Soil)	34	36.77	108.15
	DOLT-3 (Dogfish liver)	NA	NA	NA
Cd	Soil-5 (Soil)	4.3	4.714	109.62
	DOLT-3 (Dogfish liver)	NA	NA	NA

Note: NA = Not Available.

**Table 3:** Values (Mean ± SD) and ranges (min–max) of the length, width, height, fresh weight (FW) dry Weight (DW), water content in percentage (WC), condition index (CI) and conversion factor of *C. javanica.*

	PS (*n* = 106)	SM (*n* = 99)	DT (*n* = 88)	KJ (*n* = 79)	Overall (*n* = 372)
Length (mm)	14.22 ± 1.93 ^a^	17.35 ± 1.99 ^c^	15.20 ± 3.10 ^b^	15.50 ± 2.82 ^b^	15.56 ± 2.73
	10.32–19.56	12.59–22.67	11.76–24.95	10.54–22.96	10.32–24.95
Width (mm)	11.83 ± 1.57 ^a^	14.33 ± 1.64 ^c^	12.56 ± 2.42 ^b^	12.72 ± 2.18 ^b^	12.86 ± 2.16
	8.53–16.26	10.44–18.25	9.48–19.52	8.58–18.34	8.53–19.52
Height (mm)	7.78 ± 1.10 ^a^	9.44 ± 1.04 ^c^	8.12 ± 1.68 ^a,b^	8.28 ± 1.55 ^b^	8.41 ± 1.49
	5.89–11.20	6.93–12.00	6.09–13.33	5.65–12.37	5.65–13.33
Shell FW (g)	0.37 ± 0.16 ^a^	0.64 ± 0.23 ^c^	0.49 ± 0.33 ^b^	0.46 ± 0.27 ^b^	0.49 ± 0.27
	0.16–1.03	0.22–1.34	0.17–1.72	0.13–1.44	0.13–1.72
Tissue FW (g)	0.18 ± 0.08 ^a^	0.28 ± 0.12 ^b^	0.26 ± 0.18 ^b^	0.20 ± 0.11 ^a^	0.23 ± 0.13
	0.06–0.43	0.09–0.70	0.09–0.88	0.06–0.56	0.06–0.88
Shell DW (g)	0.33 ± 0.15 ^a^	0.57 ± 0.20 ^c^	0.42 ± 0.31 ^b^	0.43 ± 0.26 ^b^	0.44 ± 0.25
	0.08–0.97	0.21–1.25	0.13–1.60	0.12–1.37	0.08–1.60
Tissue DW (g)	0.02 ± 0.01 ^a^	0.03 ± 0.01 ^b^	0.03 ± 0.02 ^b^	0.03 ± 0.01 ^b^	0.03 ± 0.02
	0.01–0.05	0.01–0.07	0.01–0.11	0.01–0.08	0.01–0.11
WC Shell (%)	9.06 ± 4.83 ^b^	7.83 ± 2.69 ^a^	10.80 ± 2.55 ^c^	7.66 ± 2.56 ^a^	8.75 ± 3.65
	2.08–41.23	3.59–18.43	6.12–18.21	2.93–13.68	2.08–41.23
WC Tissue (%)	89.99 ± 2.77 ^c^	88.72 ± 2.71 ^b^	88.81 ± 2.23 ^b^	86.47 ± 3.35 ^a^	88.63 ± 3.03
	81.63–100.00	79.06–93.09	84.59–94.88	71.25–92.32	71.25–10.00
CI (g/cm^3^)	1.6 ± 0.3 ^d^	1.2 ± 0.2 ^b^	1.4 ± 0.2 ^c^	1.1 ± 0.1 ^a^	0.01 ± 0.00
	0.01–0.02	0.01–0.02	0.01–0.02	0.01–0.01	0.01–0.02
CF	0.11	0.11	0.12	0.15	

Note: Different letters indicate significantly different at *p* < 0.05.

**Table 4:** Physico-chemical parameters of the river water from the Langat River.

	PS	SD	SM	SD	DT	SD	KJ	SD
Temperature (°C)	27.42	1.25	29.23	0.87	28.91	0.35	29.55	1.30
Conductivity (mS/cm)	0.05	0.04	6.18	11.72	0.05	0.02	0.19	0.05
Conductivity (µS/cm)	39.54	11.13	42.25	66.01	48.83	16.47	209.73	56.71
Salinity	0.01	0.00	0.02	0.02	0.02	0.01	0.08	0.03
DO (mg/L)	6.49	0.94	6.95	0.49	9.11	0.44	3.35	1.48
pH	6.22	0.28	5.90	0.35	6.53	0.04	6.74	0.58

**Table 5:** Heavy metal concentrations (Mean ± Standard deviation, in µg/g dry weight) in total soft tissue of *C. javanica.* Bold numbers represent the highest concentration of heavy metal among the sampling sites.

	PS			SM			DT			KJ		
	Mean	±	SD	Mean	±	SD	Mean	±	SD	Mean	±	SD
As	2.33	±	0.87	10.50	±	8.16	2.26	±	0.43	4.33	±	2.05
Cd	0.19	±	0.01	0.26	±	0.09	0.20	±	0.07	0.21	±	0.05
Co	0.56	±	0.02	0.74	±	0.10	0.68	±	0.15	0.63	±	0.35
Cr	4.29	±	2.04	3.31	±	1.17	2.72	±	0.64	3.44	±	0.23
Cu	16.19	±	3.80	15.99	±	2.66	18.18	±	4.01	17.05	±	1.17
Fe	2335.13	±	1490.12	1523.21	±	50.13	1747.82	±	989.38	1017.92	±	308.62
Mn	4.09	±	2.55	3.79	±	1.99	2.45	±	0.60	2.96	±	0.77
Ni	13.30	±	4.69	13.69	±	4.85	10.30	±	3.18	11.55	±	1.44
Pb	10.15	±	1.81	15.79	±	8.71	17.01	±	4.04	27.39	±	14.52
Zn	180.10	±	58.09	149.42	±	26.72	141.66	±	17.80	114.30	±	20.75

**Table 6:** Concentrations of As, Cd, Co, Cr and Cu (µg/g dry weight) in molluscs from other regional studies.

	Tissue Type	As	Cd	Co	Cr	Cu	Country	Reference
*Corbicula javanica*	TST	4.51	0.22	0.65	3.45	16.85	Malaysia	This study
*Corbicula fluminea*	Tissue	-	0.3–1.6	-	-	27–50	Argentina	[57]
*Limnoperna fortune*	Tissue	-	0.4–2.1	-	-	8.7–42	Argentina	
*Corbicula fluminea*	Tissue	10.80	-	-	-	-	Portugal	[58]
*Corbicula japonica*	Gill	-	3.1–113.0	-	-	-	Russia	[60]
*Corbicula fluminea* (fw)	Gill	-	0.176	-	-	122	France	[62]
*Laternula elliptica*	Gill	-	2.9	-	0.78	8.6	Mayo Island, Antarctica	[63]
	digestive gland	-	11	-	2.1	73	Mayo Island, Antarctica	
	Kidney	-	129	-	0.9	7.3	Mayo Island, Antarctica	
*Nerita lineate*	soft tissue	5.22	3.31	-	3.12	14.9	Peninsular Malaysia	[20]
*Anodonta sp.*	Viscera	-	0.20–1.30	-	-	4.9–12.6	Vienna, Austria	[64]
	Gill	-	0.25–0.67	-	-	2.9–8.3	Vienna, Austria	
	Mantle	-	0.19–1.18	-	-	3.9–8.5	Vienna, Austria	
	Abductor muscle	-	0.13–0.76	-	-	0.9–12.7	Vienna, Austria	
*Unio pictorum*	Viscera	-	0.25–0.45	-	-	5.9–7.8	Vienna, Austria	[64]
	Gill	-	0.30–0.82	-	-	7.2–9.2	Vienna, Austria	
	Mantle	-	0.23–0.90	-	-	5.2–6.1	Vienna, Austria	
	Abductor muscle	-	0.15–0.64	-	-	2.5–6.5	Vienna, Austria	
*Radix ovate*	Soft bodies	-	0.97	-	-	122	Vienna, Austria	[64]
*Viviparus* sp.	Soft bodies	-	0.13	-	-	183	Vienna, Austria	[64]
*Onchidium struma*	Hepatopancreas	-	1.36–2.23	-	11.43–16.32	205.71–375.84	Yangtze Estuary, China	[61]
	Muscle	-	0.33–0.50	-	5.54–6.91	38.43–57.41	Yangtze Estuary, China	
	Albumen gland	-	2.15–3.16	-	4.72–5.69	50.00–86.53	Yangtze Estuary, China	
	Vitelline gland	-	4.06–4.62	-	1.23–1.67	182.64–255.66	Yangtze Estuary, China	
	Digenetic gland	-	1.47–1.97	-	5.07–9.98	140.27–166.43	Yangtze Estuary, China	
*Telescopium telescopium*	Soft tissue	-	-	-	-	49.22	Peninsular Malaysia	[56]
*Perna viridis*	TST	-	0.51–1.22	-	-	6.31–20.1	Peninsular Malaysia	[44]
*Donax trunculus*	Edible parts	1.528	0.0053	-	0.245	-	Italy	[3]
*Villorita cyprinoides*	Muscle tissue	-	1.06	23.25	-	3.58	Cochin backwaters, India	[65]

**Table 7:** Concentrations of Fe, Mn, Ni, Pb and Zn (µg/g dry weight) in molluscs from other regional studies.

	Tissue Type	Fe	Mn	Ni	Pb	Zn	Country	Reference
*Corbicula javanica*	TST	1831	3.32	12.12	18.87	146.31	Malaysia	This study
*Corbicula fluminea*	Tissue	-	-	-	-	117–163	Argentina	[57]
*Limnoperna fortune*	Tissue	-	-	-	-	48–133		
*Corbicula fluminea*	Tissue	-	-	-	-	-	Portugal	[48]
*Corbicula japonica*	Gil	-	-	-	-	-	Russia	[60]
*Corbicula fluminea* (fw)	Gill	-		-		16	France	[62]
*Laternula elliptica*	Gill	1100	18	-	0.7	107	Mayo Island, Antarctica	[63]
	Digestive gland	856	7.4	-	1.4	120		
	Kidney	3150	240	-	68	2650		
*Nerita lineata*	Soft tissue	548	-	-	18.82	111.43	Peninsular Malaysia	[20]
*Anodonta sp.*	Viscera	-	-	-	0.10–1.99	81–225	Vienna, Austria	[64]
	Gill	-	-	-	1.09–21.30	317–862		
	Mantle	-	-	-	0.16–3.18	111–328		
	Abductor muscle	-	-	-	0.12–0.98	71–269		
*Unio pictorum*	Viscera	-	-	-	0.33–0.57	132–160		[64]
	Gill	-	-	-	1.13–4.68	376–430		
	Mantle	-	-	-	0.29–2.20	142–276		
	Abductor muscle	-	-	-	0.38–0.88	145–213		
*Radix ovate*	Soft bodies	-	-	-	1.5	104		
*Viviparus* sp.	Soft bodies	-	-	-	1.37	195		
*Onchidium struma*	Hepatopancreas	235–376	1.00–6.97	-	1.23–1.95	21.73–62.81	Yangtze estuary, China	[61]
	Muscle	38.43–57.41	1.01–3.52	-	0.93–1.04	6.94–11.07		
	Albumen gland	50.00–86.53	0.86–3.73	-	0.78–0.98	18.34–36.45		
	Vitelline gland	183–256	0.27–1.11	-	0.92–1.28	5.08–35.16		
	Digenetic gland	140–166	0.29–0.96	-	0.55–0.83	19.39–36.78		
*Telescopium telescopium*	Soft issue	-	-	-	4.7	47.33	Peninsular Malaysia	[46]
*Perna viridis*	TST	-	-	-	2.0–8.76	69.40–128.90	Peninsular Malaysia	[44]
*Donax trunculus*	Edible part	-	4.255	0.327	0.071	7.625	Italy	[3]
*Villorita cyprinoides*	Muscle tissue	18,532.44	-	10.56	3.05	48.45	Cochin backwaters, India	[65]

**Table 8:** Trace metal concentrations (converted into µg/g wet weight, Mean ± SD of total soft tissue (TST) of *Corbicula javanica* from the present study (*n* = 76).

	PS ^a^	SM ^b^	DT ^c^	KJ ^d^
Zn	19.81 ± 6.39	17.93 ± 3.21	15.6 ± 1.96	17.2 ± 3.11
Cu	1.78 ± 0.42	1.92 ± 0.32	2.00 ± 0.44	2.56 ± 0.18
Pb	1.12 ± 0.20	1.89 ± 1.05	1.87 ± 0.44	4.11 ± 2.18
Ni	1.96 ± 1.39	1.64 ± 0.58	1.13 ± 0.35	1.73 ± 0.22
Fe	257 ± 164	183 ± 6.02	192 ± 109	153 ± 46.3
Mn	0.45 ± 0.28	0.45 ± 0.24	0.27 ± 0.07	0.44 ± 0.12
Co	0.06 ± 0.00	0.09 ± 0.01	0.07 ± 0.02	0.09 ± 0.05
Cr	0.47 ± 0.22	0.40 ± 0.14	0.30 ± 0.07	0.52 ± 0.08
As	0.26 ± 0.10	1.26 ± 0.98	0.25 ± 0.05	0.65 ± 0.31
Cd	0.03 ± 0.02	0.03 ± 0.01	0.02 ± 0.01	0.05 ± 0.05
Conversion factor	0.11	0.11	0.12	0.15

^a^ PS = Pangsun; ^b^ SM = Semenyih; ^c^ DT = Dusun Tua; ^d^ KJ = Kajang.

**Table 9:** Guidelines on heavy metals for food safety set by different organizations or countries. All values are presented in µg/g wet weight basis.

	Cd	Cu	Pb	Zn	Cr	As	Ni
Australia New Zealand Food Standard Code [68], Australia and New Zealand.	2	NA	2	NA	NA	1	NA
Food adulteration (metallic contamination) regulation [69], Hong Kong SAR, China.	2	NA	6	NA	1	1.4	NA
Commission Regulation (EC) No 1881/2006 [70], European Union.	1.0	NA	1.5	NA	NA	NA	NA
USFDA/CFSAN; ISSC [71], United States of America	4	NA	1.7	NA	13	86	80
Food safety and standard (contaminants, toxins and residues) regulations 2011 [72], India.	1.5	30	2.5	50	NA	NA	NA
Malaysian Food Regulation [73], Malaysia	1.00	30.0	2.00	100	NA	1.0	1.5

Note: NA = not available.

**Table 10:** Estimated daily intakes (EDI, µg/kg wet weight/day) target hazard quotients (THQ), oral reference doses (RfD, µg/kg wet weight/day) and provisional tolerable weekly intake (PTWI, µg/kg wet weight/day) of heavy metals due to consumption of *C. javanica* by average -level molluscs (ALM) and high-level mollusc (HLM) consumers in Malaysia.

		As	Cd	Co	Cr	Cu	Fe	Mn	Ni	Pb	Zn	Total THQ
PS	EDI_ALM_	0.008	0.010	0.018	0.141	0.530	76.5	0.134	0.583	0.332	5.89	
	THQ_ALM_	0.027	0.010	0.001	0.047	0.013	-	0.001	0.029	0.095	0.02	0.242
	EDI_HLM_	0.015	0.020	0.037	0.281	1.059	153	0.267	1.165	0.664	11.8	
	THQ_HLM_	0.050	0.020	0.001	0.094	0.026	-	0.002	0.058	0.190	0.039	0.481
SM	EDI_ALM_	0.038	0.009	0.027	0.118	0.571	54.4	0.135	0.489	0.564	5.34	
	THQ_ALM_	0.127	0.009	0.001	0.039	0.014	-	0.001	0.024	0.161	0.018	0.395
	EDI_HLM_	0.075	0.019	0.053	0.236	1.142	109	0.271	0.978	1.127	10.7	
	THQ_HLM_	0.250	0.019	0.002	0.079	0.029	-	0.002	0.049	0.322	0.036	0.786
DT	EDI_ALM_	0.007	0.006	0.022	0.089	0.595	57.2	0.080	0.337	0.557	4.64	
	THQ_ALM_	0.023	0.006	0.001	0.030	0.015	-	0.001	0.017	0.159	0.015	0.267
	EDI_HLM_	0.015	0.013	0.044	0.178	1.190	114	0.160	0.674	1.114	9.27	
	THQ_HLM_	0.050	0.013	0.001	0.059	0.030	-	0.001	0.034	0.318	0.031	0.538
KJ	EDI_ALM_	0.019	0.015	0.028	0.154	0.761	45.5	0.132	0.516	1.223	5.10	
	THQ_ALM_	0.063	0.015	0.001	0.051	0.019	-	0.001	0.026	0.349	0.017	0.543
	EDI_HLM_	0.039	0.031	0.056	0.307	1.522	90.8	0.264	1.031	2.445	10.2	
	THQ_HLM_	0.130	0.031	0.002	0.102	0.038	-	0.002	0.052	0.699	0.034	1.089
RfD		0.30	1.00	30.0	3.00	40.0	NA	140	20.0	3.50	300	
PTWI		15	7		23.3	500			12	25	2100	

Note: As was estimated as 10% of total As [67]. PS = Pangsun; SM = Semenyih; DT = Dusun Tua; KJ = Kajang; ALM = Average level mollusc consumer (17.86 g/day consumption); HLM = High level mollusc consumer (35.7 g/day consumption).

**Table 11:** Comparisons of estimated daily intake (EDI, µg/kg wet weight/day) values of the current study with those reported in the literature.

	Consumption Rate	EDI_As_^a^	EDI_Cd_	EDI_Co_	EDI_Cr_	EDI_Cu_	EDI_Fe_	EDI_Mn_	EDI_Ni_	EDI_Pb_	EDI_Zn_	Country	Reference
*C. javanica*	17.86 (ALM)	0.018	0.010	0.024	0.13	0.614	58.4	0.120	0.481	0.669	5.24	Malaysia	This study
	35.70 (HLM)	0.036	0.021	0.048	0.25	1.228	117	0.241	0.962	1.338	10.48		
*Nerita lineata*	17.86 (ALM)	0.032	0.205	-	0.19	0.921	-	-	-	1.164	6.89	Malaysia	[16]
	35.70 (HLM)	0.065	0.409	-	0.39	1.844	-	-	-	2.328	13.8		
*Ruditapes decussates*	36.03	-	1.062	1.523	2.71	-	-	-	0.411	4.164	-	Egypt	[83]
*Mactra coralline*	36.03	-	0.553	1.069	1.38	-	-	-	0.133	1.933	-		
*Paphia undulate*	36.03	-	0.465	0.914	1.21	-	-	-	0.023	1.605	-		
*Coralliophila meyendorff*	36.03	-	1.637	0.830	1.02	-	-	-	0.150	1.809	-		
*Venerupis rhomboids*	36.03	-	0.044	0.884	0.30	-	-	-	1.457	0.782	-		
*Crista pectinata*	36.03	-	0.024	0.269	0.33	-	-	-	0.552	0.789	-		
*Ostrea plicatula*	38.90	-	0.206	-	0.69	-	-	-	-	0.130	-	China	[84]
*Ruditapes philippinarum*	38.90	-	0.082	-	1.47	-	-	-	-	0.093	-		
*Sinonovacula constricta*	38.90	-	0.033	-	0.90	-	-	-	-	0.133	-		
*Tegillarca granosa*	38.90	-	0.228	-	1.76	-	-	-	-	0.130	-		
*Crassostrea angulata*		-	2.901	-	-	781	-	-	-	-	995	China	[86]
*Crassostrea hongkongensis*		-	1.727	-	-	3281	-	-	-	-	1865		
*Donax trunculus* ^b^	227g(Adult = 70 kg)	0.148	0.017	-	0.79	-	-	13.8	1.060	0.230	24.7	Italy	[3]
	114g(Children = 16 kg)	0.326	0.038	-	1.75	-	-	30.3	2.330	0.506	54.3		

^a^ As was estimated as 10% of total As [67]. ALM = Average level mollusc consumer (17.86 g/day consumption); HLM = High level mollusc consumer (35.7 g/day consumption). ^b^ Samples of the referred study were purchase in Catania fish market, Italy.

**Table 12:** Comparisons of target hazard quotient (THQ, unitless) values of the current study (averaged value of all four sampling sites) with those reported in the literature.

	Consumption Rate	THQ_As_^a^	THQ_Cd_	THQ_Co_	THQ_Cr_	THQ_Cu_	THQ_Fe_	THQ_Mn_	THQ_Ni_	THQ_Pb_	THQ_Zn_	Country	Reference
*Corbicula javanica*	17.86 (ALM)	0.06	0.01	0.00	0.04	0.02	0.00	0.02	0.19	0.02	0.06	Malaysia	This study
	35.70 (HLM)	0.12	0.02	0.00	0.08	0.03	0.00	0.05	0.38	0.03	0.12		
*Nerita lineata*	17.86 (ALM)	0.106	0.206		0.063	0.024	-	-	-	0.292	0.029	Malaysia	[20]
	35.70 (HLM)	0.217	0.408		0.130	0.046	-	-	-	0.581	0.046		
*Ruditapes decussates*	36.03	-	0.29	0.02	0.25	-	-	-	0.02	0.28	-	Egypt	[83]
*Mactra coralline*	36.03	-	0.15	0.02	0.13	-	-	-	0.01	0.13	-		
*Paphia undulate*	36.03	-	0.13	0.01	0.11	-	-	-	0.00	0.11	-		
*Coralliophila meyendorff*	36.03	-	0.45	0.01	0.09	-	-	-	0.01	0.12	-		
*Venerupis rhomboids*	36.03	-	0.01	0.01	0.03	-	-	-	0.07	0.05	-		
*Crista pectinata*	36.03	-	0.01	0.00	0.03	-	-	-	0.03	0.05	-		
*Ostrea plicatula*	38.90	-	0.20	-	0.22	-	-	-	-	0.04	-	China	[84]
*Ruditapes philippinarum*	38.90	-	0.08	-	0.48	-	-	-	-	0.03	-		
*Sinonovacula constricta*	38.90	-	0.03	-	0.28	-	-	-	-	0.04	-		
*Tegillarca granosa*	38.90	-	0.22	-	0.56	-	-	-	-	0.04	-		
*Crassostrea angulata*		-	2.90	-	-	19.50		-	-	-	3.32	China	[86]
*Crassostrea hongkongensis*		-	1.73	-	-	82.0		-	-	-	6.22		
*Donax trunculus* ^b^	227 g (Adult = 70 kg)	0.5	-	-	-	-	-	-	-	-	-	Italy	[3]
	114 g (Children = 16 kg)	1.1	-	-	-	-	-	-	-	-	-		

^a^ As was estimated as 10% of total As [67]. ^b^ Samples of the referred study were purchase in Catania fish market, Italy.

## Data Availability

Not applicable.
